# PACAP-38 and sex hormones in women with migraine: exploratory analysis of a cross-sectional, matched cohort study

**DOI:** 10.1186/s10194-024-01804-4

**Published:** 2024-06-11

**Authors:** Elisabeth Storch, Lucas H. Overeem, Maria Terhart, Mira P. Fitzek, Kristin S. Lange, Uwe Reuter, Bianca Raffaelli

**Affiliations:** 1 Department of Neurology, Charité Universitätsmedizin Berlin, Corporate Member of Freie Universität Berlin and Humboldt-Universität Zu Berlin, Charitéplatz 1, Berlin, 10117 Germany; 2 Clinician Scientist Program, Berlin Institute of Health at Charité (BIH), Berlin, Germany; 3 Universitätsmedizin Greifswald, Greifswald, Germany

**Keywords:** Migraine, PACAP-38, Sex hormones, Menstruation, Women’s health

## Abstract

**Background:**

Endogeneous and exogeneous sex hormones can impact the frequency and severity of migraine attacks, but the underlying mechanisms are poorly understood. In this study, we investigate the relationship between female sex hormones and Pituitary Adenylate Cyclase-Activating Polypeptide-38 (PACAP-38) concentrations in plasma of women with migraine and healthy controls, aiming to elucidate potential hormonal influences on PACAP dynamics and their relevance to migraine pathophysiology.

**Methods:**

This analysis is part of a cross-sectional, matched-cohort study. We recruited two groups of women with episodic migraine: one with a regular menstrual cycle (M-RMC) and another undergoing combined oral contraceptive treatment (M-COC). Additionally, we included corresponding age-matched control groups without migraine for both categories (C-RMC and C-COC). For participants with a RMC, the study visits were scheduled during the perimenstrual period (menstrual cycle day 2 ± 2) and periovulatory period (day 13 ± 2). Participants using COC were examined at day 4 ± 2 of the hormone-free interval and between day 7–14 of the hormone intake phase. During these visits, PACAP-38 concentrations in plasma were measured using a commercial Enzyme-linked-immunosorbent assay (ELISA) kit.

**Results:**

The study included 120 women, with 30 participants in each group. Women with migraine and a RMC had significantly higher PACAP-38 plasma concentrations compared to healthy controls at both study visits [day 2 ± 2: M-RMC: 2547.41 pg/ml (IQR 814.27 – 4473.48) vs. C-RMC: 1129.49 pg/ml (IQR 257.34 – 2684.88), *p* = 0.025; day 13 ± 2: M-RMC: 3098.89 pg/ml (IQR 1186.29 – 4379.47) vs. C-RMC: 1626.89 (IQR 383.83 – 3038.36), *p* = 0.028]. In contrast, PACAP-38 levels were comparable between migraine and control groups receiving COC. Women with migraine and a RMC exhibited higher PACAP-38 concentrations during menstruation compared to those using COC during the hormone-free interval.

**Conclusion:**

Systemic PACAP-38 concentrations in women vary based on the presence of migraine diagnosis and their hormonal status.

## Background

Migraine is a disabling neurological disorder that disproportionately affects women with a prevalence three times higher than that in men [1]. In addition, women experience a longer attack duration and a greater incidence and severity of associated symptoms, resulting in a higher overall disease burden [2].

Fluctuations of sex hormones can influence the course of migraine. During the physiological menstrual cycle, attacks occurring during the perimenstrual period are particularly frequent, long and severe [3–5]. The peak in migraine incidence following the decline in estrogen concentrations has given rise to the so-called "estrogen withdrawal hypothesis," stating that a rapid reduction in circulating estrogen concentrations can act as a trigger for migraine attacks [6].

The use of exogeneous sex hormones, such as hormonal contraception, has shown variable effects on migraine depending on the formulation, dosage and treatment regimen [7]. In women taking a combined oral contraception (COC), hormonal withdrawal during the hormone-free interval is associated with an exacerbation of migraine attacks [8, 9].

Although the influence of endogenous and exogenous sex hormones on migraine is evident, the precise mechanisms translating hormonal fluctuations into migraine attacks remain largely elusive [10]. Recent studies have pointed to an estrogen-dependent modulation of the Calcitonin Gene-Related Peptide (CGRP) pathway as a pivotal molecular pathway in migraine pathophysiology [11]. Increased CGRP release after the premenstrual estrogen decline may contribute to the increased susceptibility for migraine attacks during the perimenstrual period [4]. Supporting this hypothesis, the parent study of this analysis pointed to increased CGRP concentrations in plasma and tear fluid of women with migraine during menstruation compared to healthy controls [12]. However, evidence deriving from other studies is conflicting [13, 14]. While a hormone-dependent modulation of the CGRP pathway could contribute to explain some aspects of migraine in women, additional molecular pathway may also be implicated [15, 16].

Here, we focus on Pituitary Adenylate Cyclase-Activating Polypeptide (PACAP) as another key player in migraine pathophysiology and potential target for therapeutic interventions [17–22]. PACAP exists in various isoforms, with the 38-amino acids PACAP-38 being the predominant one [23]. PACAP and its receptors are expressed in the trigeminovascular system [24]. The PACAP-38 isoform has been shown to dilate extracerebral arteries and trigger headache in healthy individuals as well as migraine-like attacks in individuals with migraine [21, 25, 26]. In addition to its established role in migraine, PACAP influences various physiological processes within the endocrine and reproductive system [27]. This includes the regulation of gonadotropic hormones in the pituitary gland [28], and the modulation of follicular maturation and ovulation in female ovaries [29]. However, it remains unclear whether sex hormones might influence PACAP synthesis or release, and whether women with migraine exhibit different patterns compared to those without migraine.

Therefore, in this study, we aimed to evaluate the relationship between sex hormones and the systemic PACAP-38 concentrations in women with migraine under different hormonal conditions and to compare them with those of healthy women.

## Methods

### Study design and participants

This is an exploratory analysis of a cross-sectional, matched-cohort study conducted at the Headache Center, Department of Neurology, Charité Universitätsmedizin Berlin, Berlin, Germany. Detailed information about the study design and the study population can be found in the primary endpoint report [12].

For this analysis, we considered two groups of female participants with episodic migraine (EM). The first group consisted of women with EM and a regular menstrual cycle (M-RMC), characterized by a cycle duration of 28 ± 2 days. The second group consisted of women with EM undergoing treatment with a COC (M-COC) in a 21/7 regimen, defined as 21 days of hormone intake (HI) followed by a 7-day hormone-free interval (HFI). Two corresponding age-matched female groups without migraine were recruited as controls (C-RMC and C-COC).

Key inclusion criteria for the EM groups encompassed a minimum of three migraine days in the month preceding screening and a diagnosis of menstrually-related migraine according to the third edition of the International Classification of Headache Disorders criteria [30]. Participants diagnosed with pure menstrual migraine and those undergoing preventive migraine treatment were excluded from the study. Further exclusion criteria included pregnancy, lactation, current or history of gynecological and neurological disorders, as well as any medical condition requiring daily medication.

### Study procedures

The study consisted of three visits: an initial screening visit and two study visits. During the screening visit, we assessed the inclusion and exclusion criteria for study participation. This evaluation included a semi-structured interview for the participants' medical history and a physical examination.

**Fig. 1:**
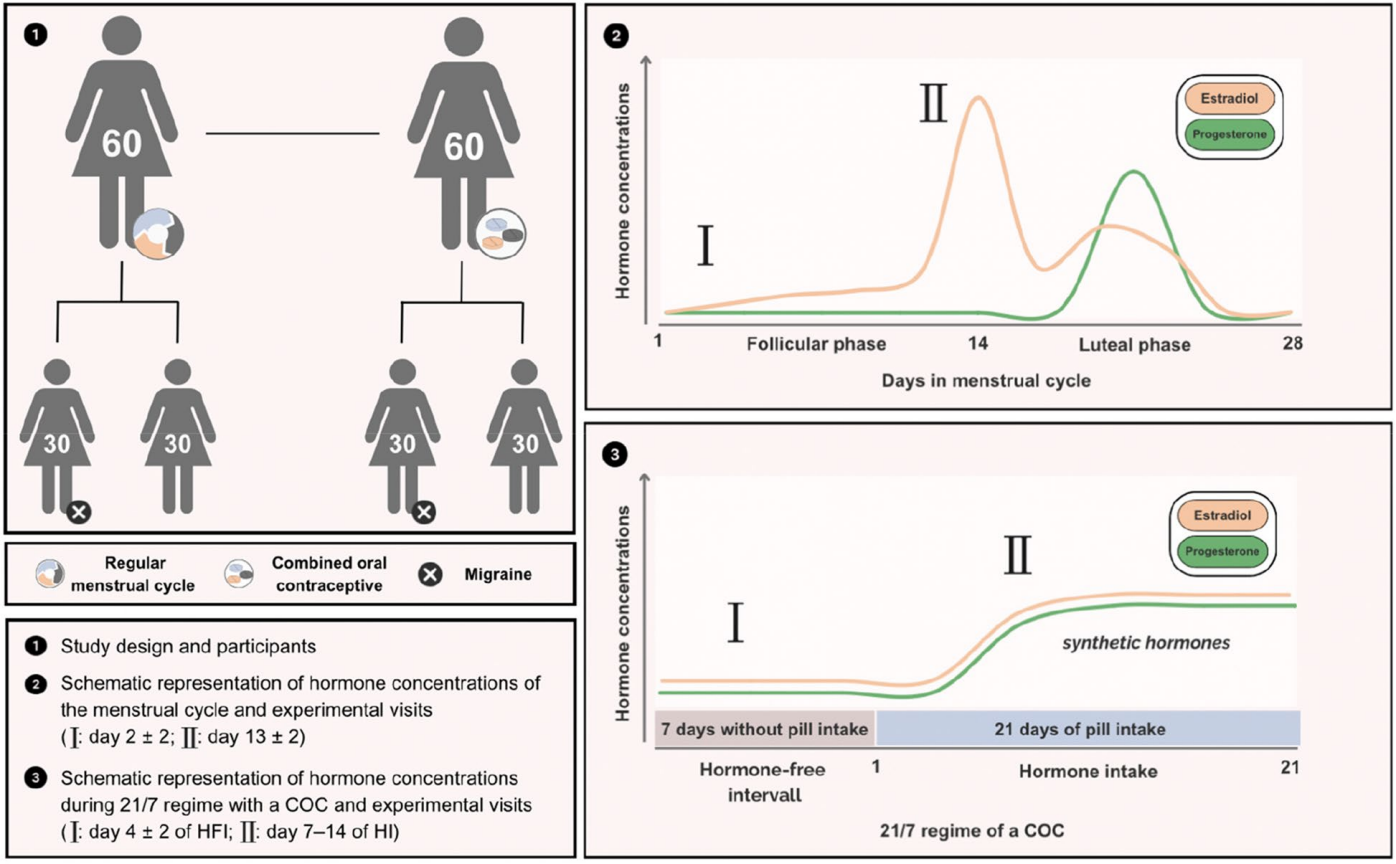
Study design with participants and scheduled study visits (created with Canva Inc.) Schematic representation of hormone levels during the menstrual cycle and under treatment with a combined contraceptive, adapted from [10, 31]

For the groups with a RMC (M-RMC and C-RMC), the study visits were scheduled on day 2 ± 2 of the menstrual cycle (perimenstrual) and on day 13 ± 2 of the menstrual cycle (periovulatory). Participants using COC (M-COC and C-COC) were examined at day 4 ± 2 of the HFI and between days 7–14 of HI (Fig. 1).

The study visits for the migraine groups occurred during the interictal period, defined as the absence of any migraine symptoms and the abstention from acute pain medication for a minimum of 12 h before and after study visits. The occurrence of a migraine attack or the use of acute medication within 12 h before or after the visit led to the visit being rescheduled or repeated.

At the study visits, participants presented at our headache center between 9 AM and 5 PM in a non-fasting state. For the analysis of PACAP-38, we collected blood using precooled 3 mL EDTA tubes (BD Vacutainer) with the addition of the protease inhibitor aprotinin (150 µl, 3–7 trypsin inhibitor unit (TIU)/mL) (Sigma Aldrich, Munich, Germany). Subsequently, we centrifuged the tubes for 15 min at -6 °C and 2,000 rpm. The plasma was then transferred to 1.5 mL polypropylene tubes (Eppendorf, Hamburg, Germany) and stored at -80 °C.

PACAP-38 concentrations in plasma were measured using a commercial sandwich Enzyme-linked Immunosorbent Assay (ELISA) kit (Wuhan Fine Biotech Co.) with a detection range of 7.813—500 pg/mL and a sensitivity of < 4.688 pg/mL. This kit was developed for the quantitative determination of PACAP-38 in serum, plasma, tissue homogenates, and other fluids of humans and exhibits good intra-assay and inter-assay reliability (coefficient of variation < 8% and < 10%). The ELISA was conducted following the manufacturer's instructions in a 1:20 dilution.

To determine sex hormone levels (estradiol, progesterone), we collected blood in 5-mL serum tubes (BD Vacutainer) at room temperature. These samples were subsequently sent to our partner laboratory (Labor Berlin, Charité Vivantes GmbH) and assessed using electrochemiluminescence immunoassay.

### Endpoints

Key endpoint of this analysis was the differences in PACAP-38 concentrations in plasma (pg/mL) between M-RMC and C-RMC at both study visits. Further endpoints were the differences in PACAP-38 concentrations in plasma between M-COC and C-COC during HFI and HI.

Additionally, we conducted a comparative analysis of PACAP-38 concentrations in plasma within the migraine groups (M-RMC and M-COC) during the perimenstrual period and HFI, as well as during the periovulatory state and HI. We also performed similar comparisons within the respective healthy control groups (C-RMC and C-COC).

Moreover, we evaluated the intraindividual correlations of PACAP-38 concentrations between both visits and correlations of PACAP-38 with estradiol and progesterone as well as the previously assessed CGRP concentrations in plasma [12].

### Statistical analysis

Sample size calculation was based on the primary analysis of the study and resulted in 30 participants per group [12]. No specific sample size calculation was performed for this analysis given its exploratory character. With an assumed effect size (d) of 0.7, and significance level set at 0.05, this sample size would provide statistical power of 80% for the primary endpoint analysis using a Mann- Whitney *U* test. We report descriptives as median and interquartile ranges (IQRs) for numerical variables and frequencies and percentages for categorical variables. Since data was non-normally distributed, we used the Mann-Whitney *U* test and Kruskal- Wallis analysis of variance to compare results between the groups. Correlation analyses were tested with Spearman rank correlations. A *p*-value below 0.05 was considered statistically significant. Statistical analysis was performed with SPSS Statistics 27 (IBM Corp., Armonk, NY).

### Ethical approvals

This study was approved by Charité Ethical Committee (EA1/004/20). Participants gave written consent following study information. This study is reported in accordance with the “Strengthening the Reporting of Observational Studies in Epidemiology” (STROBE) statement for cohort studies.

### Disclaimer

The authors of this study recognize sex and gender as distinct concepts. However, since this study included exclusively female participants who self-identified as women, we chose to use the term “women” throughout the manuscript. We recognize that the findings of this study may not be applicable to all individuals who identify as women.

## Results

The study cohort comprised a total of 120 women, 30 in each group. Demographic characteristics and sex hormone concentrations were similar between the migraine groups and the respective healthy control groups (Tables 1 and 2).

**Table 1:** Demographic characteristics of study population

	M-RMC	C-RMC	M-COC	C-COC
**Age **[**y**]	26.5	26.0	25.0	27.0
	(24.0-30.0)	(24.0-31.0)	(22.8-30.0)	(22.8-31.0)
**BMI**	22.7	20.8	22.0	20.7
	(20.0-26.6)	(19.6-22.6)	(20.0-25.0)	(20.2-23.0)
**Duration of menstrual cycle [d]**	28	28		
	(27-30)	(26-30)		
**Estradiol dose in COC [mg]**			0.03	0.03
			(0.03-0.03)	(0.03-0.03)
**Progesterone dose in COC [mg]**			2.0	2.0
			(0.2-2.0)	(0.2-2.0)
**Monthly migraine days [d]**	4.0		5.8	
	(3.9-6.3)		(4.0-7.0)	
Aura* [**n,%**]	11, 36.7 %		17, 43.3%	

*Abbreviations: C *control participants without migraine, *COC *combined oral contraception, *IQR *interquartile range, *M *women with migraine, *RMC *regular menstrual cycle. Values are median *(IQR)*

*Patients diagnosed with migraine with aura also experienced migraine attacks without aura. The number (%) indicates those who have had more than two attacks with aura in their lifetime

**Table 2:** Sex hormones concentrations in women with RMC and COC

**RMC**	**Perimenstrual**		**Periovulatory**	
	**M-RMC**	**C-RMC**	**M-RMC**	**C-RMC**
**Day of menstrual cycle**	3	2.5	14	14
	(2-4)	(2-3)	(13-15)	(12.8-15)
**LH [U/L]**	5.6	5.6	12.4	15.4
	(4.2-6.5)	(4.0-7.3)	(7.5-32.0)	(10.7-30.7)
	*p* = 0.717		*p* = 0.478	
**Estradiol [pmol/l]**	136.5	135.0	576.5	607.5
	(118.8-175.8)	(99.9-169.3)	(303.0-961.3)	(320.7-1010.9)
	*p* = 0.438		*p* = 0.830	
**Progesterone [nmol/l]**	0.8	0.85	0.85	0.95
	(0.4-1.1)	(0.5-1.3)	(0.4-2.4)	(0.5-2.7)
	*p* = 0.411		*p* = 0.544	
**COC**	**Hormone-free interval**		**Hormone intake**	
	**M-COC**	**C-COC**	**M-COC**	**C-COC**
**Day**	3	3	10	10
	(2-4)	(3-4)	(8-12)	(9.8-12.0)
**LH [U/L]**	3.2	1.7	2.6	2.2
	(0.4-5.3)	(0.3-4.2)	(1.2-4.5)	(0.3-4.9)
	*p* = 0.218		*p* = 0.264	
**Estradiol [pmol/l]**	47.7	21.9	38.0	21.3
	(20.3-99.7)	(18.4-58.0)	(18.4-65.2)	(18.4-58.0)
	*p* = 0.061		*p* = 0.438	
**Progesterone [nmol/l]**	0.3	0.3	0.4	0.4
	(0.2-0.5)	(0.2-0.6)	(0.2-0.5)	(0.2-0.7)
	*p* = 0.836		*p* = 0.267	

*Abbreviations:* *C *control participants without migraine, *COC *combined oral contraception, *IQR *interquartile range, *M *women with migraine, *RMC *regular menstrual cycle. Values are median *(IQR)*

### Women with a regular menstrual cycle: migraine vs. healthy control

**Fig. 2:**
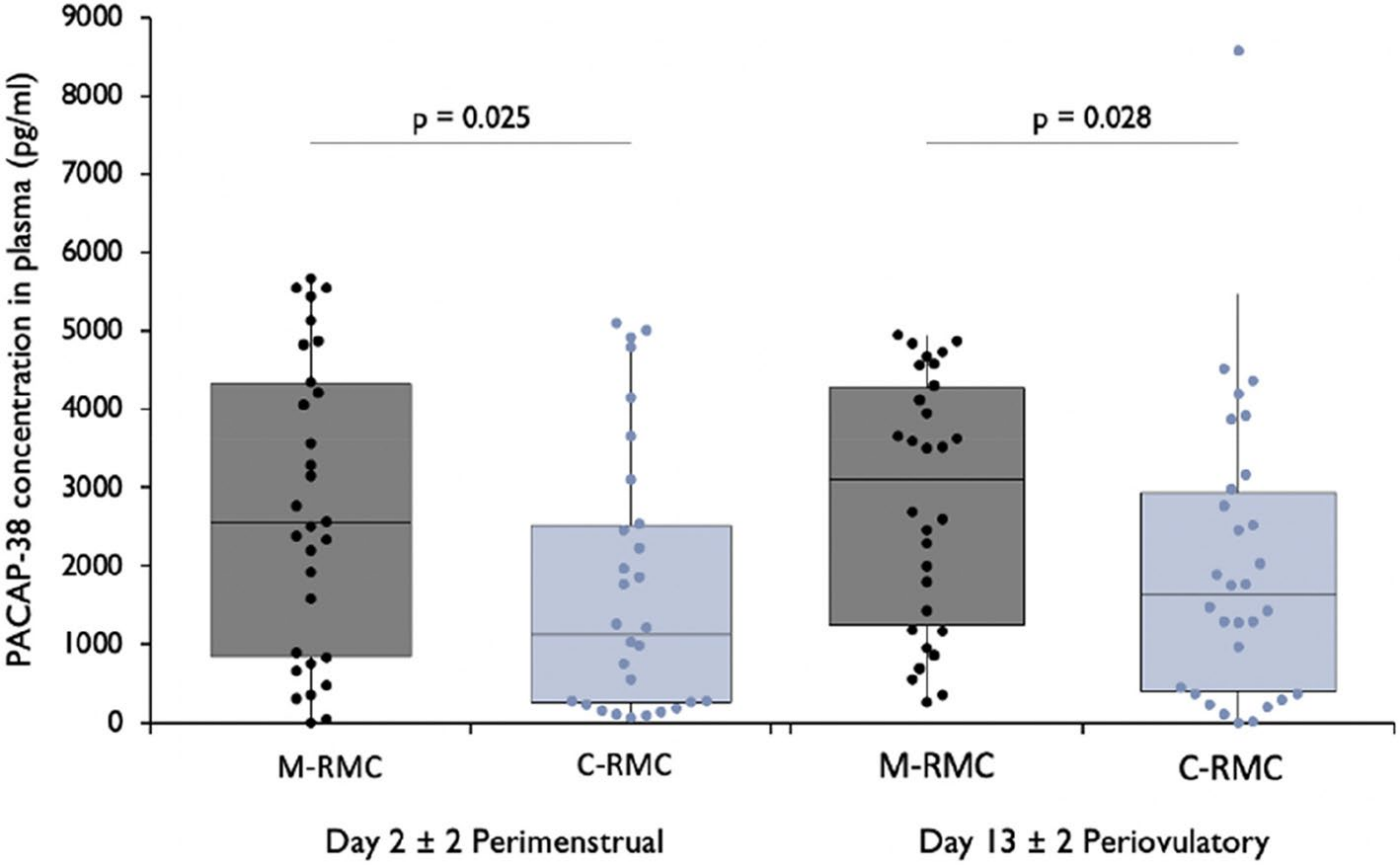
PACAP-38 concentrations in plasma in patients with migraine (M) and healthy controls (C) with a regular menstrual cycle (RMC)

Women in the M-RMC group had significantly higher PACAP-38 plasma concentrations at both study visits compared to the C-RMC group [day 2 ± 2: M-RMC: 2547.41 pg/ml (IQR 814.27 – 4473.48) vs. C-RMC: 1129.49 pg/ml (IQR 257.34 – 2684.88), *p* = 0.025; day 13 ± 2: M-RMC: 3098.89 pg/ml (IQR 1186.29 – 4379.47) vs. C-RMC: 1626.89 (IQR 383.83 – 3038.36) *p* = 0.028, Fig. 2].

There was a strong intraindividual correlation between the PACAP-38 concentrations in the perimenstrual and the periovulatory visits in M-RMC (rho = 0.592, *p* < 0.001) and the C-RMC (rho = 0.622, *p* < 0.001).

There was no significant correlation between PACAP-38 concentrations and the concurrent measurements of estradiol and progesterone or CGRP in plasma at the respective visits (Table 3, at the end of the document, insert at this point of the text).

**Table 3:** Spearman`s Correlation of PACAP-38 with estradiol, progesterone and CGRP

	**Day 2 ± 2**			**Day 13 ± 2**		
	**Perimenstrual**			**Periovulatory**		
**RMC**	**Estradiol**	**Progesterone**	**CGRP**	**Estradiol**	**Progesterone**	**CGRP**
**M-RMC PACAP-38 **	rho = 0.110, *p* = 0.562	rho = 0.036, *p* = 0.850	rho = 0.035, *p* = 0.852	rho = -0.234, *p* = 0.214	rho = 0.015, *p*= 0.937	rho = 0.006, *p* = 0.975
**C-RMC PACAP-38**	rho = 0.098, *p* = 0.606	rho = -0.006, *p* = 0.975	rho = 0.341, *p* = 0.065	rho = 0.240, *p* = 0.202	rho = 0.211, *p* = 0.263	rho = 0.018, *p* = 0.923
	**Day 4 ± 2**			**Days 7-14**		
	**Hormone-free interval**			**Hormone intake**		
**COC**	**Estradiol**	**Progesterone**	**CGRP**	**Estradiol**	**Progesterone**	**CGRP**
**M-COC PACAP-38**	rho = 0.188, *p* = 0.319	rho = -0.284, *p* = 0.128	rho = 0.229, *p* = 0.222	rho = -0.226, *p* = 0.231	rho = 0.198, *p* = 0.295	rho = -0.087, *p* = 0.646
**C-COC PACAP-38**	rho = 0.157, *p* = 0.406	rho = -0.119, *p* = 0.532	rho = -0.179, *p* = 0.343	rho = -0.079, *p* = 0.679	rho = -0.072, *p* = 0.705	rho = -0.160, *p* = 0398

*Abbreviations: C* control participants without migraine, *COC *combined oral contraception, *M *women with migraine, *RMC *regular menstrual cycle

### Women with a combined oral contraception: migraine vs. healthy control

PACAP-38 levels were similar between the M-COC and the C-COC group at the time of HFI and HI (Table 4).

**Table 4:** PACAP-38 concentrations in plasma in M-COC and C-COC

	**Day 4 ± 2**		**Days 7-14**	
	**Hormone-free interval**		**Hormone intake**	
	**M-COC**	**C-COC**	**M-COC**	**C-COC**
**PACAP-38 (pg/ml)**	1504.2	1151.8	1695.7	1328.7
	(742.1-2615.2)	(488.7-3168.6)	(691.7-3328.4)	(370.5-3219.3)
	*p* = 0.918		*p* = 0.544	

*Abbreviations:C *control participants without migraine, *COC *combined oral contraception, *IQR *interquartile range, *M* women with migraine. Values are median *(IQR)*

Our analysis showed an intraindividual correlation between the PACAP-38 concentrations in the HFI and the HI study visits in both M-COC (rho = 0.758, *p* < 0.001) and the C-COC group (rho = 0.493, *p* = 0.006). There was no significant correlation between the levels of PACAP-38 and the concentrations of estradiol and progesterone in plasma or the previously measured CGRP levels (Table 3).

### Comparison of PACAP-38 levels in women with migraine: RMC vs. COC

**Fig. 3:**
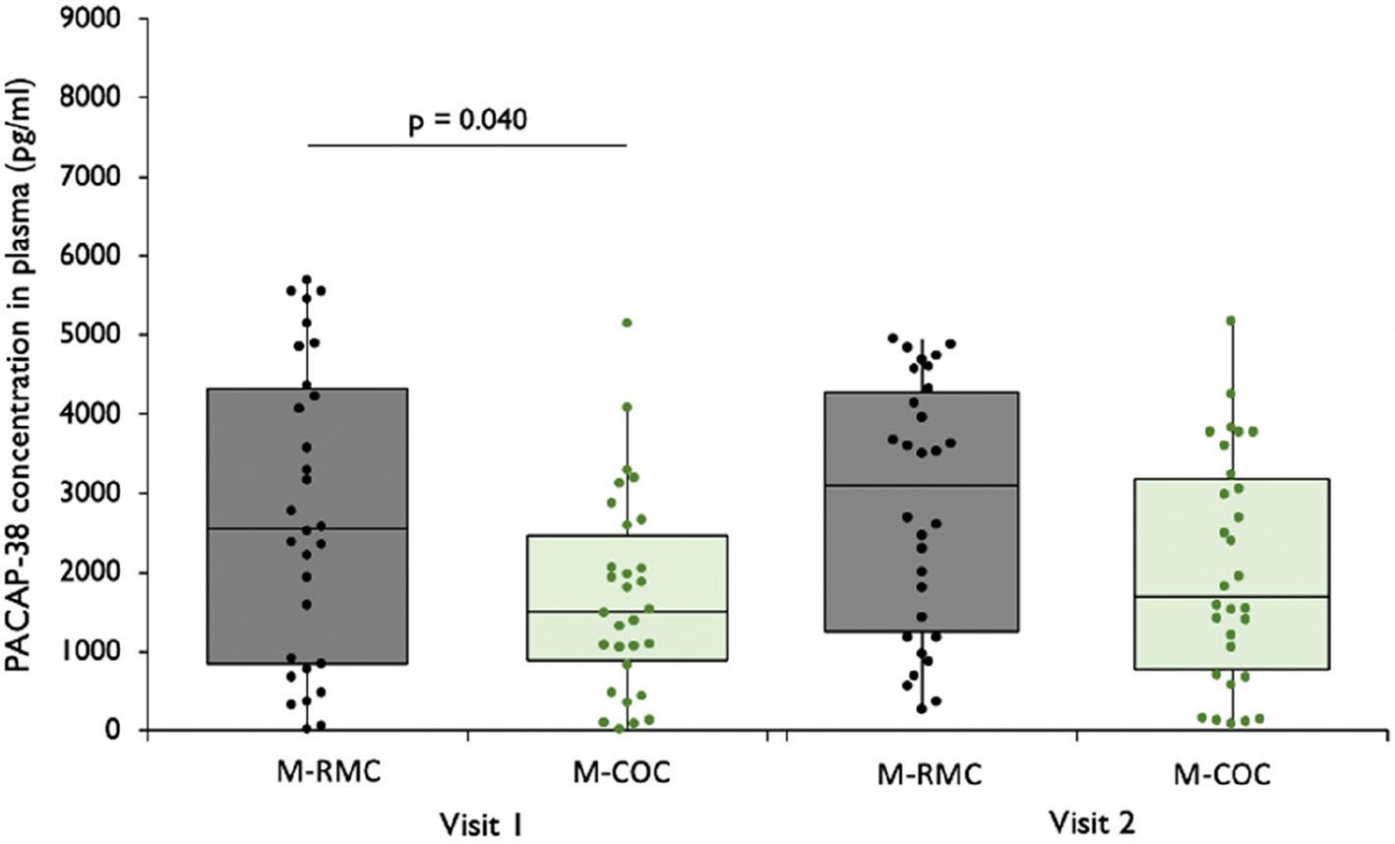
PACAP-38 in plasma in patients with Migraine (M) with a regular menstrual cycle (RMC) vs. with a combined oral contraceptive (COC). Visit 1 = day 2 ± 2 of menstrual cycle for M-RMC and day 4 ± 2 of HFI for M-COC; Visit 2 = day 13 ± 2 of menstrual cycle for M-RMC and days 7–14 of HI for M-COC

M-RMC women had significantly higher PACAP-38 concentrations in plasma during menstruation compared to M-COC women during the HFI (*p* = 0.040). In the periovulatory phase, M-RMC women displayed numerically higher PACAP-38 concentrations than M-COC women during HI, without reaching statistical significance (*p* = 0.056, Fig. 3).

### Comparisons of PACAP-38 levels in healthy participants: RMC vs. COC

There were no differences in PACAP-38 concentrations within the subgroups of healthy controls with RMC and a COC [(day 2 ± 2 vs. HFI, *p* = 0.430); (day 13 ± 2 vs. HI,

*p* = 0.853)].

## Discussion

This study shows that systemic PACAP-38 concentrations in women may vary depending on the presence of migraine and on their hormonal status. Specifically, women with migraine and a RMC had higher interictal PACAP-38 concentrations across the menstrual cycle compared to healthy control participants. In contrast, women with migraine under COC treatment had similar PACAP-38 concentrations to those observed in healthy women (C-RMC and C-COC). Among women with migraine, PACAP-38 concentrations during the physiological menstruation were higher compared to the endometrial bleeding from COC withdrawal during HFI, which might point to a distinct impact of endogeneous and exogeneous sex hormones on PACAP-38 release.

Prior investigations into interictal PACAP concentrations in patients with migraine have yielded conflicting results. For instance, Cernuda-Morollón and colleagues reported no differences in total PACAP levels in plasma between women with migraine and healthy controls [32]. Two recent pediatric studies observed higher concentrations of PACAP-38 in plasma in children with migraine during the interictal period compared to their healthy counterparts [33, 34]. In contrast, some studies detected lower PACAP concentrations in interictal patients with migraine compared to healthy individuals [35, 36]. These contradictory findings may stem from differences in study populations, definitions of the interictal period, and measurement protocols [37]. Furthermore, none of these previous studies specifically evaluated the hormonal status of participants. Tuka et al. described similar interictal PACAP-38 concentrations in women with migraine reporting a “menstruation cycle sensitivity” compared to patients without, but they did not consider their specific hormonal profiles [36]. Our findings highlight the importance of considering the specific hormonal status when evaluating interictal PACAP-38 levels. Indeed, in our cohort, only women with a RMC, but not those undergoing COC treatment, exhibited elevated interictal PACAP levels. This underscores the intricate relationship between hormonal fluctuations and PACAP dynamics in women with migraine, emphasizing the need for nuanced consideration in future research endeavors.

To date, only one study unrelated to migraine has examined PACAP plasma levels in women with various hormonal profiles, including women with a RMC, during pregnancy, after menopause, and with hormonal disorders including premature ovarian failure (POF) and idiopathic hypogonadotropic hypogonadism (IHH) [38]. Women with a RMC were assessed during menstruation, while women with POF and IHH received COC treatment and were measured during the HFI. In this previous study, PACAP levels during the physiological menstruation were significantly higher than during the HFI, which closely mirrors our findings in the migraine groups, but not in the healthy groups. However, the comparability of our findings with those of this previous study is limited due to two main factors. First, it is unknown whether the women in the previous study had migraine or other headache disorders, making it challenging to assess how this potential confounding factor may have influenced the results. Second, the women with COC in the previous study had pathological conditions, namely POF and IHH, which inherently disrupt sex hormone levels. Future studies should aim to replicate our findings in populations specifically diagnosed with migraine, while also considering other hormonal statuses such as pregnancy or postmenopause.

In our cohort, we did not observe any significant correlation between the absolute concentrations of sex hormones and those of PACAP-38. This suggests that hormonal fluctuations, rather than the absolute hormone concentrations, may exert influence on PACAP-38 levels in plasma. While serial measurements throughout the menstrual cycle present methodological challenges, such an approach would offer valuable insights into the impact of hormonal changes on PACAP-38 concentrations. Furthermore, the absence of correlation between PACAP-38 and CGRP plasma levels, which were measured in our parent study, hints at potentially distinct hormone-dependent modulation of these pathways [12]. Nevertheless, despite the lack of correlation, the overall patterns of plasma PACAP-38 and CGRP levels exhibited similarities across groups and visits. Specifically, higher concentrations of both neuropeptides were consistently observed in women with migraine and a RMC, particularly during menstruation, while lower concentrations were noted in women with migraine undergoing COC treatment and in healthy controls. This observation underscores the need for a dedicated study to investigate the intricate relationship between PACAP-38, CGRP, and fluctuating sex hormones.

The measurement of plasma PACAP-38 levels is subject to several methodological limitations that warrant consideration when interpreting our findings. The wide array of preanalytical and measurement methods reported in the literature poses challenges for direct comparison with previous studies. However, our findings show comparable concentrations in plasma to previous studies involving children with migraine [34], patients with heart failure [39], and both pregnant and non-pregnant healthy women [40].

Moreover, it remains unclear what proportion of PACAP-38 in plasma originates from the trigeminovascular system versus other sources [41]. Given the multitude of processes influencing PACAP concentrations, it is difficult to ascertain whether these dynamics are specific to migraine or due to other confounding factors. Additionally, similar to CGRP, PACAP-38 has a short half-life in plasma (approximately 5 min) and is rapidly degraded by endopeptidases [41, 42]. In our preanalytical phase, efforts were made to stabilize PACAP-38 through rapid removal and storage as well as the addition of protease inhibitors [43]. Given the exploratory nature of our study, the sample size calculation was based on the original publication [12], and its applicability to PACAP-38 analysis may require further validation in future studies.

A further limitation of our study pertains to the definition of the interictal period, set as 12 h free of migraine, which is shorter than in other investigations and may introduce bias [44]. Nevertheless, pragmatically, extending the migraine-free period would have increased the likelihood of visit cancellations. Furthermore, while the peak incidence of migraine attacks occurs at day -1 of the menstrual cycle [45], our perimenstrual visits took place slightly later. This scheduling was a result of ensuring interictal measurements and practical considerations, such as participants informing us on the first day of menstruation and being unable to visit immediately. An earlier perimenstrual timepoint might provide different insights and should be considered in future studies. In addition, we did not specifically inquire about the presence or absence of aura during the perimenstrual attacks in patients who have had more than two attacks with aura in their lifetime and thus fulfilled the ICHD-3 criteria for migraine with aura. This could influence the interpretation of our results [46] and should be considered in future research. Moreover, we cannot precisely determine whether periovulatory visits occurred on the day of ovulation or in the days immediately preceding or following it. Future studies should incorporate ovulation tests to measure the LH surge and confirm the timing of ovulation.

In conclusion, this exploratory study provides evidence of a complex interplay between PACAP-38 and sex hormones in women with migraine. The elevated PACAP-38 levels observed in participants with migraine and a regular menstrual cycle might contribute to the higher susceptibility to migraine in menstruating women.

These findings underscore the need for further longitudinal research to elucidate the role of PACAP-38 in migraine pathophysiology and its modulation by hormonal changes.

## Data Availability

Data are available from corresponding author upon reasonable request.

## Abbreviations

C-COC: healthy controls with a combined oral contraceptive
CGRP: calcitonin gene-related peptide
COC: combined oral contraceptive
C-RMC: healthy controls with a regular menstrual cycle
EM: episodic migraine
ELISA: enzyme-linked-immunosorbent assay
HFI: hormone-free interval
HI: hormone intake
IHH: idiopathic hypogonadotropic hypogonadism
IQR: interquartile range
M-COC: participants with episodic migraine and a combined oral contraceptive
M-RMC: participants with episodic migraine and a regular menstrual cycle
PACAP: Pituitary Adenylate Cyclase-Activating Polypeptide
POF: premature ovarian failure
