# Correction: PACAP-38 and sex hormones in women with migraine: exploratory analysis of a cross-sectional, matched cohort study

**DOI:** 10.1186/s10194-024-01898-w

**Published:** 2024-10-30

**Authors:** Elisabeth Storch, Lucas H. Overeem, Maria Terhart, Mira P. Fitzek, Kristin S. Lange, Uwe Reuter, Bianca Raffaelli

**Affiliations:** 1 Department of Neurology, Charité Universitätsmedizin Berlin, Corporate Member of Freie Universität Berlin and Humboldt-Universität Zu Berlin, Charitéplatz 1, 10117 Berlin, Germany; 2 Clinician Scientist Program, Berlin Institute of Health at Charité (BIH), Berlin, Germany; 3 Universitätsmedizin Greifswald, Greifswald, Germany

**Correction: J Headache Pain25**,** 98 (2024)**

10.1186/s10194-024-01804-4

This article was originally published with the incorrect Competing interests statement ‘The authors declare no competing interests’ while it should have been ‘BR is a Guest Editor of the collection Gender-Specific Aspects of Headache and is a member of the Editorial Board of The Journal of Headache and Pain. BR was not involved in the journal’s peer review process of, or decisions related to, this manuscript. The rest of the authors have no competing interests to declare’.

The original article has been updated.

